# Phage display and targeting peptides: surface functionalization of nanocarriers for delivery of small non-coding RNAs

**DOI:** 10.3389/fgene.2015.00178

**Published:** 2015-05-12

**Authors:** Babak Bakhshinejad

**Affiliations:** Department of Genetics, Faculty of Biological Sciences, Tarbiat Modares UniversityTehran, Iran

**Keywords:** phage display, peptide library, targeting, delivery, nanocarrier, small non-coding RNA

## Introduction

Small non-coding RNAs are known as a clinically relevant category of non-coding RNAs (ncRNAs) that have gained growing attention for their therapeutic values. miRNA and siRNA are of the most important and well-documented types of small ncRNAs. These regulatory RNA molecules are short–approximately 22 nucleotides long–with a similar mechanism of generation in which they are excised from longer double-stranded RNA precursor molecules. Both miRNA and siRNA target protein-coding genes via an antisense-based strategy and follow the same processing manner to reach gene-silencing effect (Chen et al., 2015). Functionally, small ncRNAs play a determining part in the modulation of cellular gene expression. The impressive role of these molecules as the controlling machinery of cells has proposed promising capacities for their potential translation into the clinic. In this context, tuning the regulatory components (such as ncRNAs) rather than the regulated components (protein-coding gens) has been suggested to be a more convenient and more effective approach to correct many cellular dysfunctions (Taft et al., 2010). This emerging concept symbolizes the dramatic potential of small ncRNAs, as a significant part of the regulatory apparatus of cells, to treat human diseases.

## Small ncRNA delivery: active targeting and the dream of magic bullet

Within the recent years, nanotechnology-based approaches have emerged as potentially powerful and efficient systems for cellular delivery of ncRNAs. The exploitation of nanocarriers shows promise to bypass a variety of biological barriers for systematically administered RNA therapeutics (Miele et al., 2012; Segovia et al., 2015). Nanoparticles provide protection from serum nucleases for RNA-based therapeutic molecules, decrease their sequestration by phagocytes of the reticulo-endothelial system (RES) and are able to carry both hydrophilic and hydrophobic substances (Aagaard and Rossi, 2007; Peer et al., 2007). The excellent characteristics of nanocarrier platforms aid in enhancing overall bioavailability, extending *in vivo* stability, and improving the cellular delivery of RNA therapeutics such as small ncRNAs.

For the successful delivery of small ncRNA, one of the most important problems that needs to be solved is targeting of these drugs into the desired sites. The delivery of small ncRNAs to the diseased cells, tissues or organs not only raises their silencing potency, but also leads to a considerable reduction of side effects by avoiding normal cells (Daka and Peer, 2012). Active targeting plays a highly critical part in achieving efficient *in vivo* biodistribution and optimized systemic delivery of ncRNA-based therapeutics. Active targeting becomes possible when the surface of nanocarriers that are embedded with small ncRNAs is modified through the covalent attachment of a cell/tissue-specific ligand. These surface-conjugated ligands will specifically interact with receptors that are expressed on the surface of target cells (Bertrand et al., 2014). In this manner, the delivery of therapeutic small ncRNA molecules will be poised to materialize the dream of constructing magic bullet; the concept that was theoretically fathered by Paul Ehrlich to describe targeted transportation of therapeutic agents to the desired sites of the body. Peptides are known as one of the most interesting categories of targeting ligands that have recently received huge attention. Due to some unique properties, peptides have become favorable targeting ligands for formulating powerful and sophisticated drug delivery platforms.

## Phage display libraries: a treasure of cell-targeting peptides

Phage display that is defined as the ability to genetically modify bacteriophages for introducing specific amino acids onto their surface is a worthwhile tool in biomedicine. The birth of phage display, as a kind of virus-based molecular chimera, goes back to three decades ago when Smith for the first time reported the expression of a foreign polypeptide on the surface of phage particles (Smith, 1985). The power of phage display for gaining momentum as a revolutionary strategy for gene/drug delivery studies comes from two distinctive features: (i) establishing a physical connection between the phenotype (the displayed ligand) and the genotype (the DNA sequence encoding the displayed ligand) within the same viral particle and (ii) producing very large libraries of ligands displayed on the surface of the phage particles. The most widely-used phage display libraries are random peptide libraries (RPL). In simple terms, a random peptide library is a collection of phages carrying on their surface extremely diverse types of peptides.

Due to the phenotype-genotype link, phage peptide libraries can be screened against any target of interest for selecting target-specific binders. This screening strategy displays similarity to the approach of natural selection but with distinctive difference of being performed in the test tube instead of nature (Bakhshinejad and Sadeghizadeh, 2014). Affinity selection is a hallmark of phage display screening through which binding peptides specific to any target including organic and inorganic structures can be obtained. The procedure of affinity-based library screening is called “panning.” Whole cells in the culture are one of the most important targets used in panning studies. Cell panning involves incubating the phage library with target cells. Some phages interact with and bind to the target with higher affinity. The unbound or weakly-bound phages are then washed away and the bound phages are recovered. Subsequently, the recovered phages are amplified through infecting host bacterial cells. This process is repeated for several rounds to ultimately isolate the most strongly binding phages whose surface displayed peptides are identified by DNA sequencing (Bakhshinejad et al., 2014).

## Phage library selected peptides and targeted delivery of miRNA/siRNA-based nanocarriers

Currently, RPLs are known to be one of the major sources of clinically relevant peptides. The marriage of combinatorial peptide chemistry and phage display has turned phage peptide libraries to a treasure for the discovery of useful targeting peptides. As we are beginning to more deeply understand the biological functions of ncRNAs in cellular processes and thereby their vital roles in the pathogenesis of numerous disorders, phage peptide libraries are attracting increasing attention for the delivery of small ncRNAs. Cell-specific peptides derived from phage libraries have been demonstrated to be capable of acting as important targeting components in nanocarriers and delivering both miRNA and siRNA into target cells. A variety of nanocarriers have been used for the delivery of small ncRNAs (Conde et al., 2015). Liposomes, polymers, micelles, protamine, and virus-like particles (VLPs) are delivery platforms that in different studies have represented potential to achieve delivery of miRNA and siRNA to target cells/tissues. Table 1 indicates a list of studies in which peptides selected by phage display screening have been used for targeted delivery of miRNA/siRNA-containing nanocarriers to desired cells.

**Table 1:** **Studies in which peptides obtained through screening of phage display libraries have been used to functionalize the surface of nanocarriers for targeted delivery of miRNA and siRNA**.

**Non-coding RNA**	**Nanocarrier**	**Targeting peptide sequence**	**Target**	**Reference**
miR-145	Branched polyethylenimine (bPEI) polymer	CRIMRILRILKLAR RIMRILRILKLARC	Breast cancer	Lee et al., 2013
PRDM14 siRNA	Liposome	DMPGTVLP (attached to M13 phage fusion protein pVIII)	Breast cancer	Bedi et al., 2011
siRNA cocktail against cyclin family members	VLPs of MS2 bacteriophage	SFSIIHTPILPL	Hepatocellular carcinoma	Ashley et al., 2011
GAPDH siRNA	Phage-like nanoparticles/Nanophage	VEEGGYIAA DWRGDSMDS	Breast cancer	Bedi et al., 2013
fluorescein-labeled siRNA	Protamine	KYLAYPDSVHIW	LNCaP – prostate cancer	Qin et al., 2011
Fas siRNA	Poly (cystamine bisacrylamide-diaminohexane, CBA-DAH) polymer	WLSEAGPVVTVRALRGTGSW	Primary cardiomyocyte	Nam et al., 2010
VEGF siRNA	Poly (cystamine bisacrylamide-diaminohexane, CBA-DAH) polymer	WLSEAGPVVTVRALRGTGSW	Cardiomyocyte	Nam et al., 2012
SHP1 and Fas siRNA	Poly (cystamine bisacrylamide-diaminohexane, CBA-DAH) polymer	WLSEAGPVVTVRALRGTGSW	Cardiomyocyte	Nam et al., 2011
miR-92a	Polyelectrolyte complex micelles	VHPKQHR	Endothelial cells	Kuo et al., 2014

Cancer is a disease that has been the focus of much investigation for the delivery of small ncRNA-based therapeutics. Peptides obtained from screening of phage libraries against various tumor cells have served to target nanocarriers with encapsulated ncRNAs to the cells of interest. In line with this, several attempts have been made to identify phage-borne peptides specific to different tumors. A number of these peptides have indicated favorable outcomes for the delivery of peptide-targeted miRNA/siRNA-containing nanocarriers to malignant cells. DS 4-3, breast cancer-specific peptides, conjugated to branched polyethylenimine (bPEI) have been indicated to deliver the tumor suppressor miR-145 to metastatic breast cancer cells, thereby inhibiting tumor cell growth and suppressing cell invasion (Lee et al., 2013). In a novel strategy for intracellular delivery of siRNA into cancer cells, a research group led by Petrenko targeted liposomes by using preselected intact phage proteins (Bedi et al., 2011). In this work, not only cancer-avid peptide but the whole fusion phage coat protein in combination with its displayed peptide was used as a targeting ligand. This tumor-specific complex (phage fusion protein and peptide) was isolated from affinity screening of a phage library against MCF-7 breast cancer cells followed by being inserted into the lipid bilayer of liposome. The result was construction of a siRNA-encapsulated phage protein-targeted liposome that specifically down-regulated the expression of PRDM14 gene–a gene with important roles in the carcinogenesis of breast cancer–and inhibited the synthesis of its protein. This strategy remarkably overcomes the problem of purification of targeting peptides and their subsequent conjugation to nanocarriers.

Phage display selected peptides have also been exploited for targeted delivery of small ncRNAs involved in disorders other than cancer. Cardiovascular diseases, as one of the leading causes of death worldwide, have attracted attention to obtain benefits from achievements of phage display technology in the development of targeted therapy approaches. Since cardiomyocyte apoptosis is an important player in several heart diseases, the inhibition of this type of cell death has been viewed as a useful strategy for potential treatment of pathological conditions linked to cardiovascular system. This can be achieved through abolishing the activity of those factors whose function stimulates apoptosis. The conjugation of a primary cardiomyocyte (PCM) specific peptide selected by phage display to a polymer-based nanocarrier was used for the delivery of siRNA specific to Fas (an apoptosis inducer) to cardiomyocytes under hypoxic conditions (Nam et al., 2010). This PCM-targeted siRNA delivery system led to the prevention of apoptosis through down-regulating the Fas gene. Sometimes the inhibitors of ncRNAs, not ncRNAs themselves, have to be delivered to target cells. The exploitation of miRNA inhibitors is a prime example of miRNA-based scenarios that can be used for therapeutic purposes. As dysregulated miRNAs are associated with some disorders, the ability to inhibit the cellular activity of these miRNAs can provide new opportunities to treat human diseases. In this context, a micelle-based nanocarrier targeted with a phage display selected peptide specific to vascular endothelial cells was shown to successfully deliver miR-92a inhibitor to endothelial cells; a strategy that is helpful for cell-specific delivery and targeted inhibition of miRNAs that contribute to the pathology of atherosclerosis (Kuo et al., 2014).

## Conclusion

The capacity of small ncRNAs in specifically silencing the expression of disease-associated genes underlines their importance as novel forms of therapeutic intervention in numerous human disorders. Nanocarriers, due to some tunable features, represent huge potential to overcome delivery issues concerned with miRNA/siRNA-based pharmaceutical platforms. Recent findings highlight the significance of phage display methodology in providing a wide variety of peptides to be used for targeting of nanocarriers to desired cells. Although still in its infancy, phage peptide libraries are receiving growing attention for targeted cellular delivery of miRNA/siRNA therapeutic cargoes. Active targeting of nanocarriers through surface modification with phage selected peptides will provide new opportunities for taking advantage of the tremendous potential of small ncRNAs in the clinical setting. The quickly advancing role played by phage peptide libraries in the territory of targeted delivery of RNA therapeutics has roots in the surprising diversity of these libraries and the convenience of their screening over a wide variety of cellular targets that cover numerous human diseases. Cell-targeting peptides derived from phage display libraries have raised hopes for researchers to take great strides toward the cherished goal of targeted delivery of miRNAs and siRNAs into the same cells that should take these therapeutic agents.

### Conflict of interest statement

The author declares that the research was conducted in the absence of any commercial or financial relationships that could be construed as a potential conflict of interest.
